# Thalamic inflammation after brain trauma is associated with thalamo-cortical white matter damage

**DOI:** 10.1186/s12974-015-0445-y

**Published:** 2015-12-01

**Authors:** Gregory Scott, Peter J. Hellyer, Anil F. Ramlackhansingh, David J. Brooks, Paul M. Matthews, David J. Sharp

**Affiliations:** Division of Brain Sciences, Department of Medicine, Hammersmith Hospital Campus, Imperial College London, London, UK; Centre for Neuroimaging Sciences, Institute of Psychiatry, Psychology and Neuroscience (IoPPN), King’s College London, London, UK; Institute of Clinical Medicine, Aarhus University, Aarhus, Denmark; Computational, Cognitive and Clinical Neuroimaging Laboratory, 3rd Floor, Burlington Danes Building, Hammersmith Hospital, Du Cane Road, London, W12 0NN UK

**Keywords:** Microglia, Translocator protein, Positron emission tomography, Traumatic brain injury, Traumatic axonal injury, PK11195, Thalamus

## Abstract

**Background:**

Traumatic brain injury can trigger chronic neuroinflammation, which may predispose to neurodegeneration. Animal models and human pathological studies demonstrate persistent inflammation in the thalamus associated with axonal injury, but this relationship has never been shown in vivo.

**Findings:**

Using [^11^C]-PK11195 positron emission tomography, a marker of microglial activation, we previously demonstrated thalamic inflammation up to 17 years after traumatic brain injury. Here, we use diffusion MRI to estimate axonal injury and show that thalamic inflammation is correlated with thalamo-cortical tract damage.

**Conclusions:**

These findings support a link between axonal damage and persistent inflammation after brain injury.

## Introduction

Traumatic brain injury (TBI) is a risk factor for dementia [1], and survivors may deteriorate years after their injury [2]. However, the mechanisms relating TBI to neurodegeneration are unclear [1]. An important factor is likely to be neuroinflammation in the form of glial activation triggered by the TBI and which can persist for many years [3]. Chronic activation of microglia is implicated in many neurodegenerative disorders [4]. Previously, using [^11^C]-PK11195 (PK) PET, a marker of the translocator protein (TSPO) expressed by activated microglia, we observed inflammation in the thalamus up to 17 years after TBI [3]. Why thalamic inflammation persists, remote from sites of focal injury, remains uncertain.

The spatial pattern of microglial activation following brain injury may relate to the white matter architecture of the CNS [5, 6]. Experimental axotomy induces microglial activation remote from the primary lesion site [5]. The thalamus is highly connected and shows chronic inflammation after central and peripheral nerve damage [5, 7]. After stroke, microglial activation is seen at the primary lesion but later emerges in projection areas including the thalamus [8, 9]. Following TBI, axons in white matter are highly susceptible to damage, making traumatic axonal injury (TAI) one of the most common pathologies of TBI [10]. Together, these observations suggest thalamic inflammation following TBI may result from the high density of connectivity between the thalamus and damaged axons.

Diffusion tensor imaging (DTI) can map the structure of thalamo-cortical white matter tracts using probabilistic tractography [11], but TBI patients with TAI pose challenges to these methods [12]. Recently, we developed a template-based approach for more robust estimation of thalamo-cortical tract integrity after TBI [12].

## Hypothesis

Here, we combine this approach with PK PET to test the hypothesis that chronic thalamic inflammation after TBI can be explained by thalamo-cortical white matter damage.

## Methods

Ten patients with a history of moderate-severe TBI (Mayo classification) (mean age ± s.d. 43 ± 5.3 years, range 36–54; mean time since injury ± s.d. 6.2 ± 5.3 years, range 0.9–17) had PK PET and structural MRI scans including DTI (see [3] for details). Two age-matched healthy control groups were used. Seven controls (mean age 46 ± 4.0, range 42–50) had PET and structural MRI. Thirteen controls (41.8 ± 6.6, 35–56) of similar premorbid intellectual ability had MRI including DTI. The project was approved by Hammersmith and Queen Charlotte’s and Chelsea Research Ethics Committee. All participants gave informed consent.

**Fig. 1:**
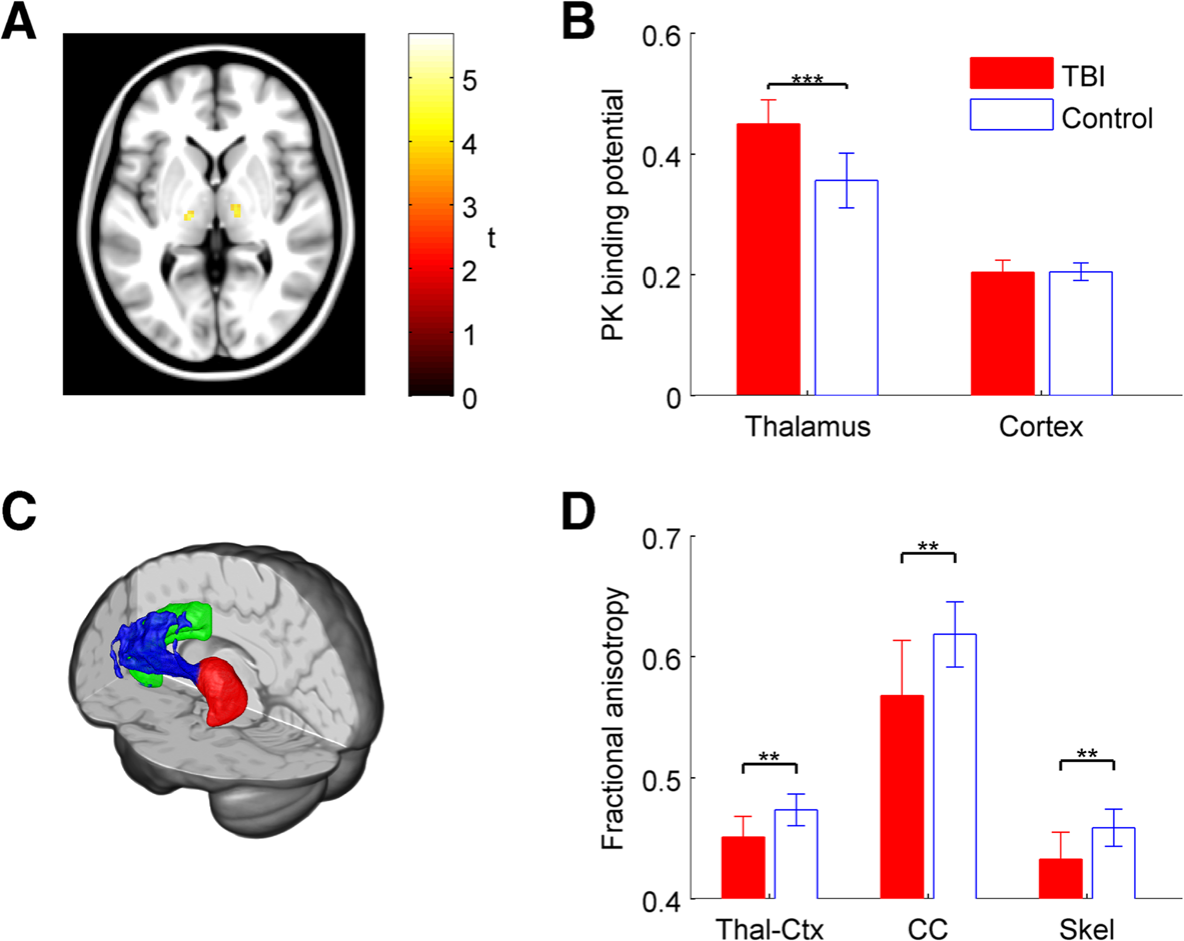
Increased thalamic microglial activation and white matter damage in TBI. **a** Statistical parametric maps (reproduced from [3]) rendered onto a standard T1 MRI image showing areas of significantly increased [^11^C]-PK11195 (PK) binding potentials (BP) in the TBI patients relative to controls. Bilateral increases in PK binding are seen in thalami. *t* values are shown. Voxels are shown significantly surpassing the voxel-wise threshold (*p* < 0.001) and the spatial extent threshold (10 voxels). Voxel-wise contrasts were performed on spatially normalised PK BP images, smoothed with a 12-mm full-width at half maximum (FWHM) Gaussian kernel, using SPM5 (see [3] for details). **b** PK BP in the thalamus and cortical grey matter, defined using anatomical regions of interest, in TBI patients (*red*) and controls. Group mean ± standard error of the mean (SEM) is shown; ***p < 0.001. **c** Tract mask (*blue*) connecting the left thalamus (*red*) to the left anterior cingulate cortex (ACC) (*green*), produced using probabilistic tractography in healthy controls (see [12]). Fractional anisotropy (FA) was sampled using bilateral thalamo-cortical tract masks. **d** FA in thalamo-cortical (*Thal-Ctx*) body of the corpus callosum (*CC*) and across the white matter skeleton (*Skel*) in TBI patients (*red*) and controls; **p < 0.01

Standard MRI T1 and DTI protocols were used (see [3]). For DTI analysis, we used a method for assessing thalamo-cortical white matter connections that is robust to the presence of TAI [12]. We combined ten thalamo-cortical tracts, previously defined in healthy controls using probabilistic tractography, into a single region of interest (ROI) (see Fig. 1c for example of one tract, and [12]). A mask of cortico-cortical tracts through the body of the corpus callosum was used as a control (from [13]), since these tracts were not connected to the thalamus. Voxel-wise maps of fractional anisotropy (FA), a measure of directionality of water flow along white matter tracts and hence their integrity, were calculated, and FA maps were skeletonised [14], leaving the central section of tracts, to minimise partial volume effects (see [12]). We calculated the mean FA of voxels within the ROIs and of all voxels in the skeleton. The B0 data for one patient contained an image artefact, so their DTI data were excluded.

To test whether thalamic PK binding is more strongly correlated to proximal than distal thalamo-cortical damage, FA was sampled from a series of non-overlapping ring-shaped ROIs extending centripetally from the thalamus from 2 to 60-mm diameter and intersected with the thalamo-cortical tract mask. ROIs were created by repeatedly dilating a thalamic mask by two voxels (2 mm). Ring-shaped ROIs were produced by subtracting the previous mask from each step.

PK PET acquisition and analysis are described in [3]. To produce maps of PK binding potential (BP_ND_), we used a supervised clustering procedure to identify reference clusters of voxels in grey matter having PK time activity curves mirroring those of controls [3]. PK BP_ND_ was sampled in the bilateral thalamus as well as the cortical grey matter regions that were used to define the thalamo-cortical tracts [12].

Regional PK BP_ND_ was compared between groups using independent sample *t* tests. Mean FA values were similarly compared. Bonferroni multiple comparisons correction was used. In TBI, we tested for a relationship between regional PK BP_ND_ and thalamo-cortical FA using linear partial correlations, controlling for age and time since injury as both factors potentially influence DTI [15] and PK binding [16].

## Results

A voxel-wise contrast showed increases in thalamic PK BP_ND_ in TBI patients versus controls (Fig. 1a, reproduced from [3]). PK binding in the thalamus ROI was significantly increased (*t* = 4.64, df = 15, *p* < 0.001, Fig. 1b). In contrast, there was no difference in cortical grey matter PK binding between the groups.

FA was decreased in TBI patients versus controls in thalamo-cortical projections (df = 20, *t* = −3.68, *p* = 0.0015), the corpus callosum (df = 20, *t* = −3.264, *p* = 0.004) and the white matter skeleton (df = 20, *t* = −3.450, *p* = 0.003) (Fig. 1d).

**Fig. 2:**
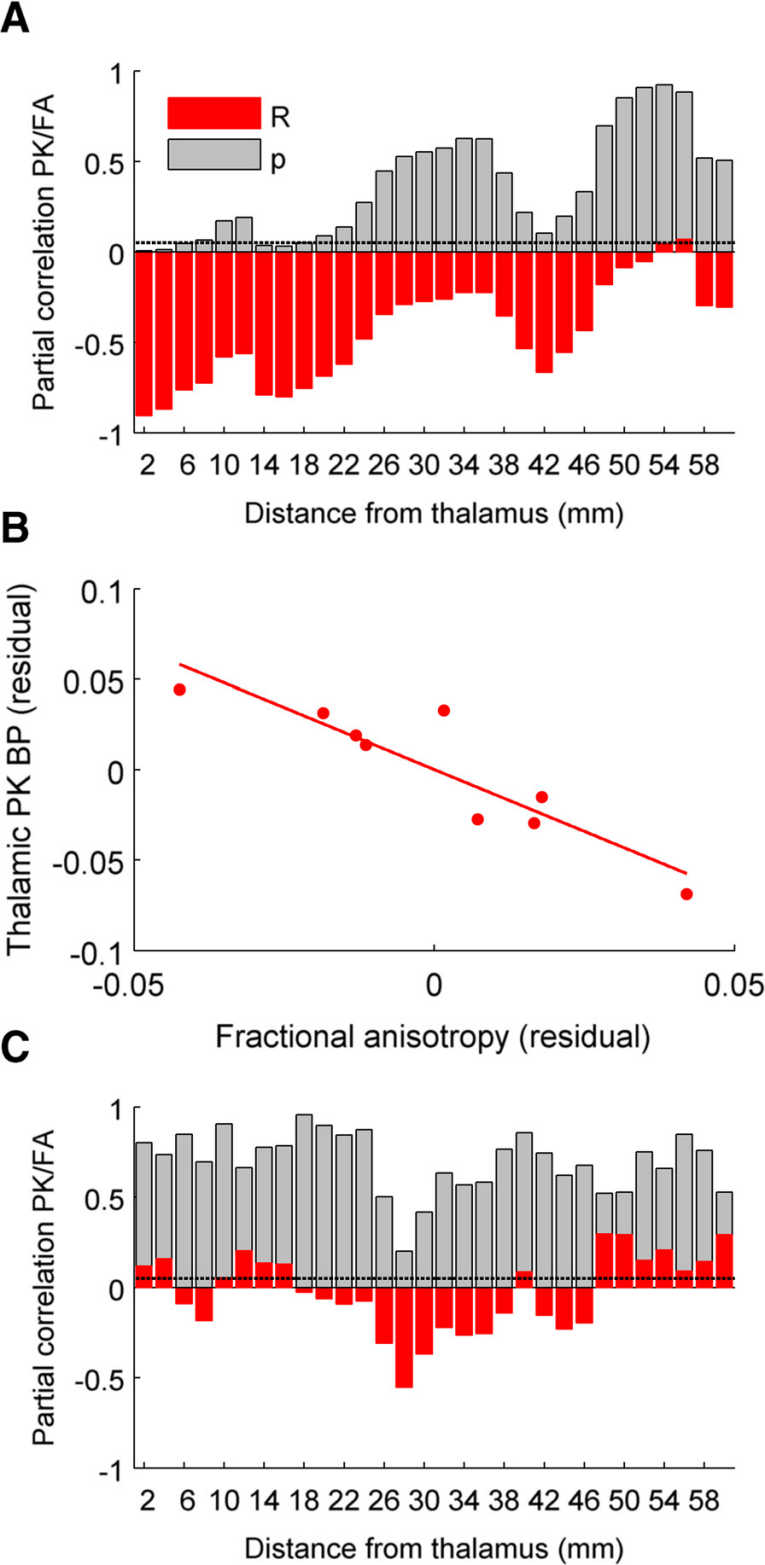
Correlation of thalamic microglial activation and white matter damage in relation to distance from thalamus. **a** Partial correlation of thalamic [^11^C]-PK11195 (PK) binding potentials (BP) and thalamo-cortical fractional anisotropy (FA), sampled with increasing distance from the thalamus in TBI patients. Distance of 0 mm reflects sampling from tracts involving the thalamus proper. R (*red*) and *p* values are shown, with a threshold of *p* = 0.05 (*dotted line*). **b** Plot of residuals after for thalamo-cortical FA (*x-axis*), sampled from a ring-shaped mask 2 mm from the outer border of the thalamus versus thalamic PK BP (*y-axis*). **c** Partial correlation of PK BP in cortical grey matter and thalamo-cortical FA, sampled and plotted as in **a**

In TBI, we found a significant negative correlation between thalamic PK BP_ND_ and mean thalamo-cortical tract FA (*r* = −0.770, *p* = 0.042). The strength of this correlation decreased with the distance at which FA was sampled from thalamo-cortical tracts (Fig. 2a). Thalamic PK binding was most strongly correlated with FA of voxels within 10 mm of the thalamus, maximally within 2-mm distance (Fig. 2b). There was no correlation between thalamic PK binding and FA of the corpus callosal tracts nor between cortical PK and thalamo-cortical FA, either when sampled as a whole or ROIs at different distances (Fig. 2c). We also found no significant correlation between time since injury and either mean thalamo-cortical FA or thalamic PK.

## Discussion

Axonal injury underlies many long-term problems after TBI [17]. Glia become activated at sites of injury [8, 18] but also at distant sites [19], including subcortical nuclei like the thalamus [18]. Our TBI patients showed both TAI and persistent thalamic microglial activation. For the first time in vivo, we show that the degrees of thalamic microglial activation and thalamo-cortical white matter tract damage are closely related.

**Fig. 3:**
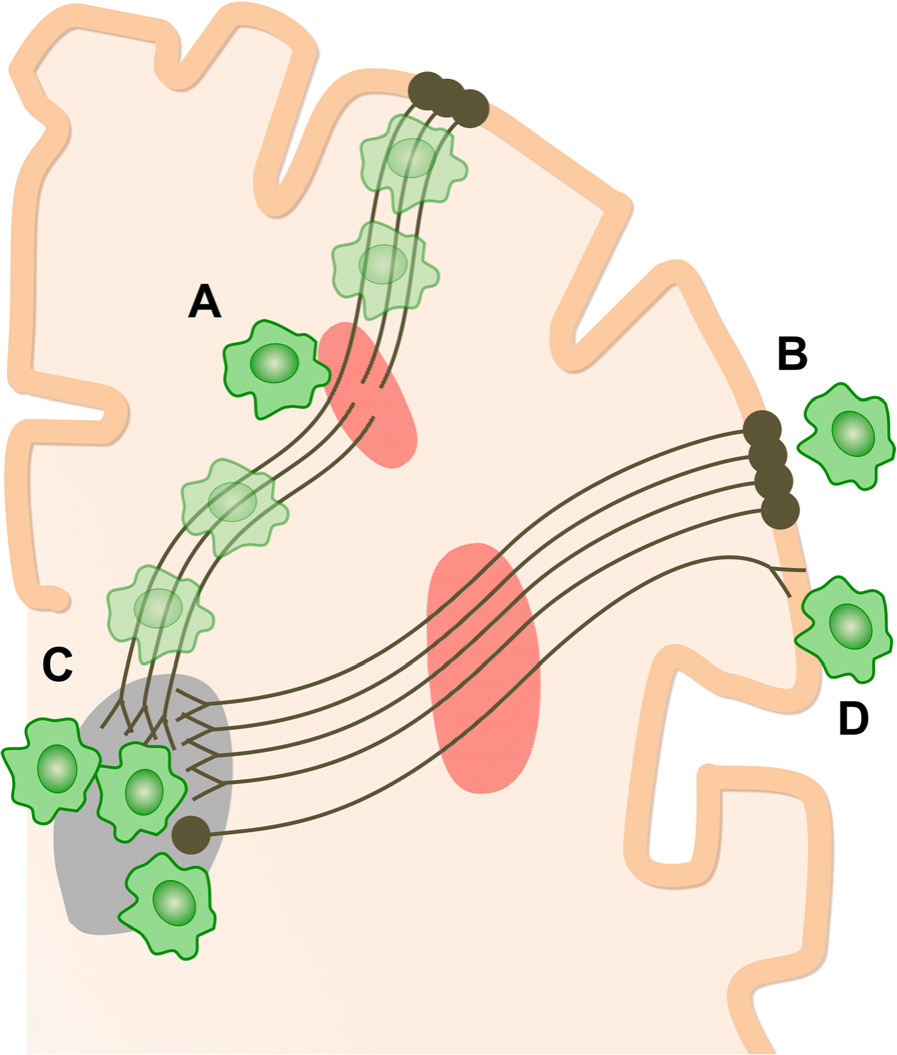
How chronic microglial activation and axonal injury may be linked after traumatic brain injury (TBI). Microglial activation (*green cells*) and traumatic axonal injury in thalamo-cortical white matter tracts (*red areas*) have been demonstrated after TBI. Sites of chronic microglial activation can co-localise with axonal abnormality (**a**) as well as along the entire axonal tract affected by injury. Remote from sites of primary axonal injury, microglia may be observed both in retrograde projection areas, towards the cell bodies of damaged neurons (**b**), and in anterograde areas (**c** and **d**). The thalamus is a highly connected structure. Thalamic microglial activation may be observed after TBI because of the high density of connections to damaged axons. The number of cortico-thalamic projections far exceeds thalamo-cortical projections. If microglial activation preferentially favours anterograde involvement, then relatively increased activation would be expected in the thalamus (**c**) compared to corresponding cortical areas (**b**)

Several mechanisms might underlie persistent thalamic inflammation after TBI (Fig. 3). It may be a response to focal injury. However, there was no MRI evidence of focal thalamic injury. Furthermore, there was no increase in PK binding in focal lesions [3], making a prolonged response to direct damage an unlikely explanation. Alternatively, thalamic inflammation may relate to a persistent effect of TAI. This is made more likely by the observation that activated microglia are seen at sites of TAI in acute and chronic phases [19]. The co-localisation of myelin basic protein immunoreactivity within microglia at sites of TAI suggests myelin fragments may provide a persistent trigger for inflammation [19]. The strong correlation we observed between thalamic PK binding and white matter close to the thalamus suggests a causative role for the persistent effects of TAI years after injury.

Following experimental axotomy, microglial activation is seen in both anterograde and retrograde projection areas [5]. Hence, an inflammatory response might be expected in cortical and subcortical projection areas following TAI. Several factors may explain the lack of PK binding in cortex versus thalamus. Firstly, cortico-thalamic projections are ~tenfold more numerous than thalamo-cortical projections [20]. This asymmetry is especially relevant if there is also a difference in the magnitude of anterograde and retrograde microglial reactions to TAI, potentially a second factor. If both factors are important, then high PK binding in the thalamus rather than cortex would suggest an anterograde, rather than retrograde, reaction predominates (Fig. 3). However, a third factor is the high density of thalamo-cortical (and cortico-thalamic) neurons in thalamus versus cortex. As Banati and colleagues observe, the high density of neurons converging in the thalamus might lead to a cumulative signal that represents a “regional amplification of … widespread but sub-threshold cortical pathology” [21].

An anterograde evolution of microglial activation is seen in other conditions. After stroke, microglial activation spreads in an anterograde fashion along damaged tracts [8, 9], and the degree of anterograde activity in the brainstem has been shown to be correlated with pyramidal tract damage. Other diseases involving white matter damage, including multiple sclerosis and peripheral nerve injury [5] and another study of TBI [22], also show thalamic inflammation, suggesting the thalamus is a common “sink” of secondary inflammation in response to axonal damage, perhaps related to its dense white matter connectivity.

Whether chronic thalamic inflammation is harmful or restorative is uncertain. This question is challenging partly because the relationship between activated microglial phenotype and TSPO expression is not clear [23]. It may be that microglia’s role in synaptic plasticity may underlie much of what we have labelled “neuroinflammation” [7, 24]. How TSPO expression and microglial activation are related has recently been addressed by Banati and colleagues, who showed using TSPO knockout mice that TSPO expression and activation of microglia following injury are not mutually dependent [25].

An important limitation of the study is that it involved a small sample of patients and with a history of a single moderate-severe TBI. There is a need to replicate these preliminary findings in larger cohorts, which might benefit from investigating a wider range of diffusion metrics. Studies from animal models of repetitive mild TBI show persistent inflammation both at sites of axonal injury and locations remote from the site of focal lesions, suggesting a similar microglial response in cases of lower injury severity [26, 27].

Evidence of persistent neuroinflammation suggests that the window of opportunity for therapeutic intervention following TBI may be longer than is usually considered. Inflammatory PET imaging provides a biomarker to investigate this prolonged inflammatory reaction and potentially the effects of interventions targeting glial activation. Our findings emphasise the need for large multi-modal longitudinal studies combining inflammatory PET with diffusion MRI to image axonal injury.

## Abbreviations

BP_ND_: binding potential
CNS: central nervous system
DTI: diffusion tensor imaging
FA: fractional anisotropy
PET: positron emission tomography
PK: [^11^C]-PK11195
ROI: region of interest
TAI: traumatic axonal injury
TBI: traumatic brain injury
